# Microbial membrane lipid adaptations to high hydrostatic pressure in the marine environment

**DOI:** 10.3389/fmolb.2022.1058381

**Published:** 2023-01-06

**Authors:** Anandi Tamby, Jaap S. Sinninghe Damsté, Laura Villanueva

**Affiliations:** ^1^ Department of Marine Microbiology and Biogeochemistry (MMB), NIOZ Royal Netherlands Institute for Sea Research, Den Burg, Netherlands; ^2^ Department of Earth Sciences, Faculty of Geosciences, Utrecht University, Utrecht, Netherlands

**Keywords:** piezophile, high hydrostatic pressure, extremophile, membrane, membrane adaptation, microbial adaptation, phospholipid

## Abstract

The deep-sea is characterized by extreme conditions, such as high hydrostatic pressure (HHP) and near-freezing temperature. Piezophiles, microorganisms adapted to high pressure, have developed key strategies to maintain the integrity of their lipid membrane at these conditions. The abundance of specific membrane lipids, such as those containing unsaturated and branched-chain fatty acids, rises with increasing HHP. Nevertheless, this strategy is not universal among piezophiles, highlighting the need to further understand the effects of HHP on microbial lipid membranes. Challenges in the study of lipid membrane adaptations by piezophiles also involve methodological developments, cross-adaptation studies, and insight into slow-growing piezophiles. Moreover, the effects of HHP on piezophiles are often difficult to disentangle from effects caused by low temperature that are often characteristic of the deep sea. Here, we review the knowledge of membrane lipid adaptation strategies of piezophiles, and put it into the perspective of marine systems, highlighting the future challenges of research studying the effects of HHP on the microbial lipid composition.

## 1 Introduction

The deep-sea is defined by a water depth of >1,000 m and encompasses most of Earth’s biosphere. At these depths, the average sea temperature is 2°C and the hydrostatic pressure is >10 MPa (100 bars) increasing by 1 MPa (10 bars) every 100 m (Jannasch and Taylor, 1984). High hydrostatic pressure (HHP) in deep-sea environments usually coincides with low bottom-water temperatures except for areas in the vicinity of hydrothermal vents and warm seas, such as the Mediterranean, and the Black Sea, which average bottom temperature are 12°C and 8°C, respectively (Lacombe et al., 1985; Vargas-Yáñez et al., 2017).

Microorganisms adapted to HHP are usually known as piezophiles, referring to their preference for high pressure (Kato, 2011). Regarding the adaptability to HHP, microorganisms can be piezolerant, if they can withstand HHP but have similar or faster growth rates at atmospheric pressure, while obligate piezophiles can only grow under HHP (Kato, 2011). Piezophiles are usually found at the bottom of the water column thus these microorganisms are both piezophiles and psychrophiles (adapted to low temperatures). The fact that these two factors coincide makes it challenging to discern the effects of HHP and low temperature on microorganisms. A strategy to pinpoint the microbial effects of HHP solely is to focus on microorganisms isolated from warmer deep-sea bottom waters or from locations close to hydrothermal vents outside of areas with extremely high temperatures. Nevertheless, to our knowledge research to pull these two effects apart are scarce (Bartlett, 1999; Nogi, 2008; Wang et al., 2009).

HHP impacts protein folding, metabolic rate, and membrane stability, leading to cell disruption. Thus, piezotolerant and piezophiles have developed various adaptive strategies to cope with HHP, such as accumulation of protein-stabilizing solutes, gene expression modulation (such as induction of genes encoding for heat shock protein), and changes in the composition of the cell membrane (Simonato et al., 2006; Oger and Jebbar, 2011).

An essential microbial cell feature that has been observed to change under HHP is the composition of the cell membrane, which represents the cell barrier against environmental stimuli. The cell membrane is a dynamic compartment composed of membrane lipids and proteins. HHP has been seen to affect membrane proteins, for example, by increasing membrane diffusion by the activation of porins (proteins forming membrane channels) (Bartlett, 1999), or by increasing the abundance of respiratory terminal oxidase able to maintain their integrity under HHP (Tamegai et al., 2011). Changes in the membrane lipid composition to keep the membrane fluid are known as homeoviscous adaptation, while the modification of the proportion of lipids in a crystalline state is referred as homeophasic adaptation (Bartlett, 2002; Fang and Bazylinski, 2008). Membrane fluidity (i.e., a parameter describing the freedom of movement of lipids and proteins within the membrane) can be adapted by modifying the degree of lipid packing, which directly affects the water permeability across the membrane. In addition, other biophysical characteristics of the cell membrane have been seen to be relevant to modulate the integrity of the membrane such as thickness, phase properties, and viscosity (Chwastek et al., 2020).

Here, we review the knowledge of membrane lipid adaptation strategies of piezotolerant and piezophile microbes encountered in the water column of marine systems. Most studies have been conducted in this setting since the deep-sea represents by far the most widespread environment where microorganisms thrive under HHP. Several structural features of the membranes have been examined, such as the composition of polar headgroups and degree of unsaturation and methylation of fatty acyl chains, and the presence of hopanoids and sterols. We conclude this review by outlining the future challenges to determine membrane lipid adaptations to HHP.

## 2 Microbial membrane lipids and their response to HHP

**FIGURE 1:**
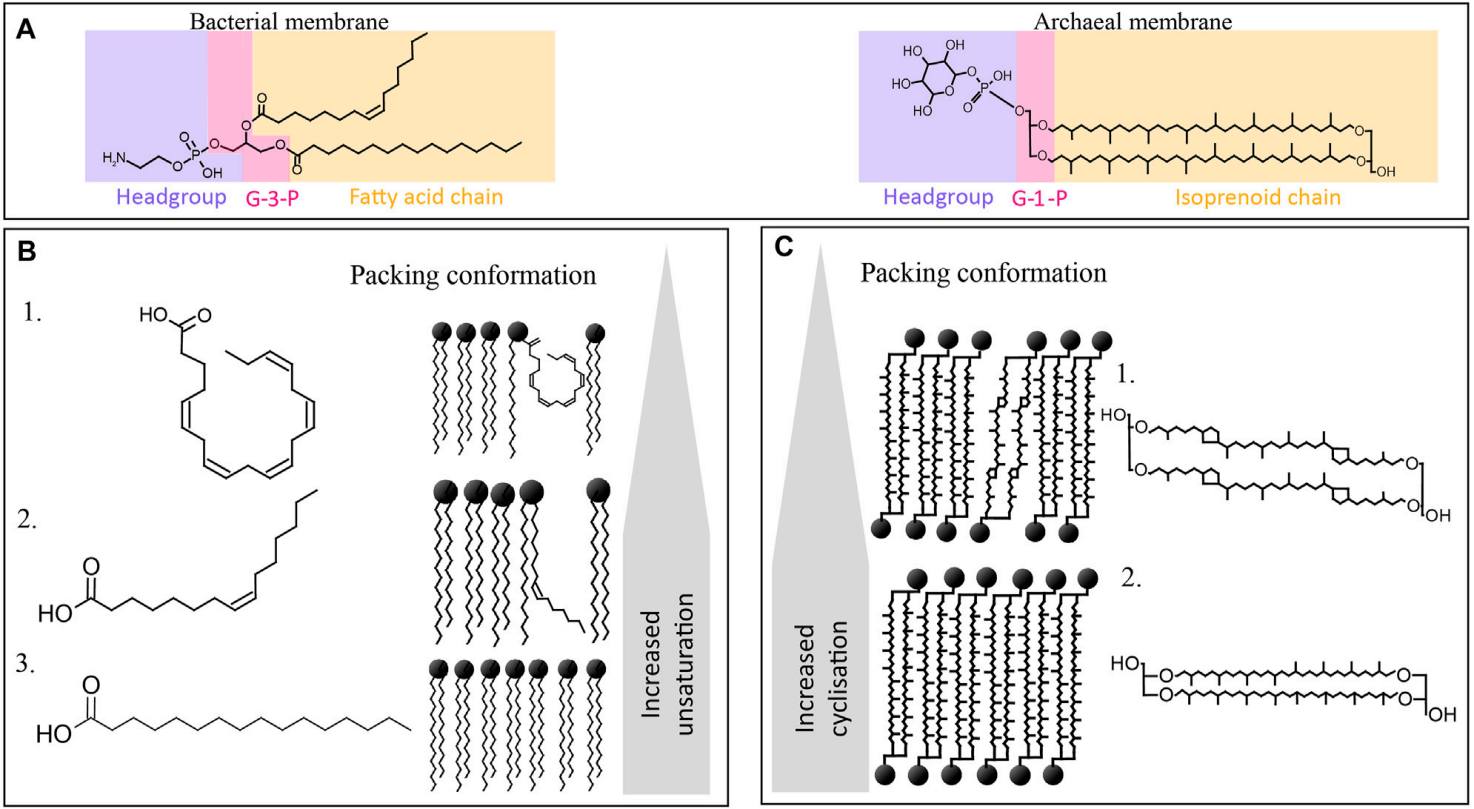
Overview of the chemical structures of bacterial and archaeal membrane lipids, including **(A)** different stereochemistry in the backbone being glycerol-3-phosphate (G3P) in bacteria and G1P in archaea (labeled in pink), and a different type of side chain (in yellow), i.e., fatty acids esterified to the G3P in bacteria, and isoprenoid alkyl chains linked through ether bonds to the G1P moiety in archaea. Polar headgroup is labeled in blue. **(B)** Different fatty acid side chains of phospholipid fatty acids (PLFA) commonly encountered in the bacterial membranes of piezophiles, with (B1) being C20:5, (B2) being (Z)-hexadec-9-enoic acid or palmitoleic acid, and (B3) being hexadecenoic acid or palmitic acid (C16), and their influence in the packing conformation and fluidity of the membrane. **(C)** Archaeal tetraether membrane lipids, being (C1) GDGT-4 with four cyclopentane moieties, and (C2) GDGT with no cyclopentane moieties (i.e., GDGT-0), and their impact on the conformation and fluidity of the membrane.

Bacteria and eukaryotes have similar adaptive strategies, as they share characteristics in the structure of their lipid membranes with their lipids composed of fatty acyl chains connected through ester bonds to glycerol-3-phosphate at positions *sn*-1 and *sn*-2, and polar head groups (Figure 1A). Both bacterial/eukaryotic and archaeal membrane lipids have mainly glycerophospholipids (GPLs) composed by a glycerol moiety, a phosphate group, and a variable head group. GPLs are polar lipids - they have an amphiphilic nature, meaning they have both a hydrophilic (with a strong affinity to water, soluble in water) and a hydrophobic (lacking affinity for water) part.

Archaeal membrane lipids differ substantially from those of bacteria and eukaryotes as they are composed of two linear isoprenoidal alkyl chains made up of a phytanyl chain (containing 20 carbon atoms, or C20) bound through ether-bonds to the *sn-*2 and *sn-*3 position of glycerol-1-phosphate (G1P) (Figure 1A). Thus, they are referred to as glycerol diether or archaeol. In addition, archaea can also form monolayers of tetraethers or so-called glycerol dibiphytanyl glycerol tetraethers (GDGTs) (Figure 1A). The isoprenoid cores can be modified by unsaturation, hydroxylation, or presence of cyclopentane or cyclohexane rings (only in the case of crenarchaeol synthesized by Thaumarchaeota) (Schouten et al., 2013).

These fundamental chemical differences between bacterial/eukaryotic and archaeal membranes lead to specific adaptation when encountering membrane-disrupting parameters such as HHP, depressurization, or extreme temperature (Siliakus et al., 2017). To adapt to HHP and extreme temperature, bacteria and eukaryotes modulate the degrees of unsaturation and branching of their acyl chain (Figure 1B) and adapt the type and proportion of polar headgroups, while archaea modulate their ratio of diether lipid to GDGT (Figure 1C).

## 3 Adaptations in the composition of bacterial/eukaryotic membrane lipids to HHP

### 3.1 Acyl chain composition

Most research on membrane lipid adaptation has focused on modulation of the fatty acyl chains of the bacterial/eukaryotic lipids in response to physical parameters. For example, saturated fatty acyl chains allow the lipids to be packed in a tighter and denser configuration than lipids with unsaturated chains, resulting in a more rigid membrane structure (Small, 1984); (Figure 1B). Monounsaturated phospholipid fatty acyl chains lead to a slight curled configuration, thus loosening the membrane, leading to an increase in fluidity (Figure 1B). In addition to unsaturation, hydroxylation (i.e., the presence of hydroxy groups), methylation, and length of the fatty acyl chain can also impact the fluidity of the membrane by impacting the melting temperature (Ernst et al., 2016).

In general, piezophiles are known to increase the level of fatty acyl chain unsaturation as an adaptive response to HHP. For instance, the model organism *Photobacterium profundum* SS9*,* a piezo- and psychrophile, has been reported to increase the unsaturation in its fatty acyl chain upon HHP as well as the bacterium *Alteromonas 4033-B* (Table 1; Kamimura et al., 1993; Allen et al., 1999). The presence of multiple unsaturations of the fatty acyl chain, or so-called polyunsaturated fatty acids (PUFAs), is also a common strategy among piezophiles. PUFAs that are most frequently associated with adaptation to HHP are C20:5 and C22:6 (Bartlett, 1999; Kawamoto et al., 2011; Usui et al., 2012; Moi et al., 2018). For example, two strains of *Psychromonas* (2D2 and 16C1) were isolated from the intestines of a deep-sea fish, *Coryphaenoides yaquinae,* which lives at 6,000 m depth. Under HHP, both strains significantly increased the content of C20:5 and C22:6, confirming that this lipid plays an important role in microbial adaptation under HHP (Table 1; Yano et al., 1998). Another example is *Shewanella piezotolerans* WP3, which increases the relative abundance of C20:5 upon HHP [Table 1; (Wang et al., 2009; Kawamoto et al., 2011)]. Surprisingly, however, the phylogenetically related piezophile *Shewanella violacea* DSS12 adopts an opposite strategy; it decreases the relative abundance of C20:5 upon HHP (Kawamoto et al., 2011).

**TABLE 1:** Summary of piezophiles and piezotolerant microorganism.

Organism	Strain number	Optimal growth pressure and temperature	Relative abundance of main FA under optimal conditions	Main change observed under HHP incubations	Reference
*Photobacterium profundum*	SS9	15**°**C—28 MPa	C16:1 (30%)	Increase of C20:5	Bartlett et al. (2014)
			C16:0 (22%)		
			C14:0 (10%)		
			C18:1 (7%)		
			C20:5 (7%)		
			*Iso* C16:0 (6%)		
			C14:1 (4%)		
*Alteromonas sp*	RS103	25**°**C**—**25 MPa	*Iso* C15:0 (30%)	Increase of unsaturated fatty acid	Kamimura et al. (1993)
			*Iso* C17:1 (20%)		
			*Iso* C17:0 (12%)		
			C16:1 (10%)		
			C18:1 (8%)		
			C16:0 (8%)		
*Psychromonas*	2D2	5**°**C—40 MPa	C16:1 (50%)	Increase content of C22:6	Yano et al. (1998)
			C22:6 (20%)		
			C14:0 (11%)		
			C16:1 (10%)		
*Psychromonas*	16C1	5**°**C—20 MPa	C16:1 (58%)	Increase content of C22:6	Yano et al. (1998)
			C16:0 (13%)		
			C14:0 (10%)		
			C22:6 (10%)		
			C14:1 (6%)		
*Shewanella piezotolerans*	WP3	15°C–20**°**C—20 MPa	C16:1 (23%)	Increase of branched fatty acid Increase of C20:5	(Xiao et al., 2007; Wang et al., 2009)
			C15:0 (12%)		
			*Iso* C13:0 (9%)		
			C18:1 (8%)		
			C20:5 (6%)		
*Shewanella violaceae*	DSS12	8**°**C—30 MPa	C16: 1 (19%)	Decrease of C20:5	Nogi et al. (1998b)
			C16: 0 (16%)		
			C20:5 (15%)		
			*Iso* C15:0 (14%)		
			C15:0 (7%)		
			C14:0 (6%)		
*Pseudomonas* sp	BT1	30**°**C—10 MPa	C18:1 (47%)	Increase of phosphatidylethanolamine (PE)	Kaneko et al. (2000)
			C16:1 (30%)		
			C16:0 (18%)		
			C18:0 (4%)		
*Psychromonas hadalis*	K41G	6**°**C—60 MPa	C16:1 (37%)	*Only cultured in optimal conditions*	Nogi et al. (2007)
			C16:0 (31%)		
			C22:6 (8%)		
*Shewanella benthica*	ATCC 43992	10**°**C—70 MPa	C16:1 (37%)	*Only cultured in optimal conditions*	Nogi et al. (1998b)
			C14:0 (17%)		
			C16:0 (15%)		
			*Iso* C13:0 (11%)		
			C20:5 (8%)		
*Moritella japonica*	JCM 10249	15**°**C—50 MPa	C16:1 (50%)	*Only cultured in optimal conditions*	Nogi et al. (1998a)
			C16:0 (21%)		
			C18:0 (18%)		
			C22:6 (6%)		
*Colwellia piezophila*	Y223G	10**°**C—60 MPa	C16:1 (50%)	*Only cultured in optimal conditions*	Nogi et al. (2004)
			C16:0 (30%)		
			C14:0 (10%)		
*Moritella yayanosii*	DB21MT-5	70**°**C—10 MPa	C16:1 (48%)	*Only cultured in optimal conditions*	Nogi and Kato (1999)
			C14:0 (15%)		
			C16:0 (13%)		
			C22:6 (9%)		
			C14:1 (6%)		
*Sporosarcina sp*	DSK25	35**°**C—0.1 MPa	*Iso* C15:0 (25%)	Increase of *anteiso-*C15:0 FA	Wang et al. (2014)
			*Anteiso* C15:0 (24%)		
			C16:1 (15%)		
			*Anteiso* C17:0 (10%)		
			*Anteiso* C17:0 (10%)		
			C16:0 (6%)		
			*Anteiso* C17:1 (4%)		
*Clostridium paradoxum*	DSM 7308	60**°**C—22 MPa	*Iso* C15:0 (65%)	Increased of branched fatty acid	Scoma et al. (2019)
			*Anteiso* C15:0 (7%)		
			C16:0 (6%)		
			C14:0 (6%)		
			*Iso* C13:0 (4%)		
*Psychromonas kaikoae*	JCM 11054	10**°**C—50 MPa	C16:1 (56%)	*Only cultured in optimal conditions*	Nogi et al. (2007)
			C16:0 (13%)		
			C14:1 (10%)		
			C14:0 (7%)		
*Archaea*					
*Thermococcus barophilus*	MP	85**°**C—40 MPa	-	Increased relative abundance of diethers	Cario et al. (2015)
*Methanococcus jannaschii*	-	85**°**C—25 MPa	GDGT-0 (35%)	Increase of macrocyclic archaeol	Kaneshiro and Clark (1995)
			Macrocyclic archeol (65%)	Decrease of GDGT-0	

Although these studies confirm that the microbial membrane can adapt to HHP by changing the relative abundance of the PUFA C20:5, other studies have shown that the presence of this PUFA is not essential to withstand HHP since, e.g., some bacterial strains such as the piezotolerant *Pseudomonas* sp. BT1, do not contain C20:5 in its membrane (Kaneko et al., 2000)**.** Moreover, the lack of production of C20:5 by mutation of specific biosynthetic genes in the piezotolerant strains *S. piezotolerans* WP3 and *S. violacea* did not impair their cell viability but only reduced their growth rate under HHP conditions suggesting the synthesis of PUFA is not a requirement to withstand HHP (Wang et al., 2009; Usui et al., 2012). Both strains compensated for the absence of C20:5 by increasing the relative abundance of monounsaturated fatty acids (MUFAs), supporting the fact that both PUFAs and MUFAs are involved in the membrane adaptation to HHP (Table 1). This is further supported by other studies, showing that mutant strains of *P. profundum* with a deficit in C20:5 could withstand both low temperature and high pressure, while mutants with reduced C18:1 were unable to grow under those conditions (Allen et al., 1999). The membrane of piezophilic and piezotolerant microorganisms harbors also other types of PUFAs, such as the C22:6 (Table 1), which typically occurs in the piezophilic and piezotolerant strains of the genera *Moritella* and *Colwellia* (DeLong and Yayanos, 1986; Oger and Cario, 2014), as well as *Psychromonas hadalis, Psychromonas* strain 2D2*,* and strain 16C1 (Table 1; Yano et al., 1998; Nogi et al., 2007)**.**

**FIGURE 2:**
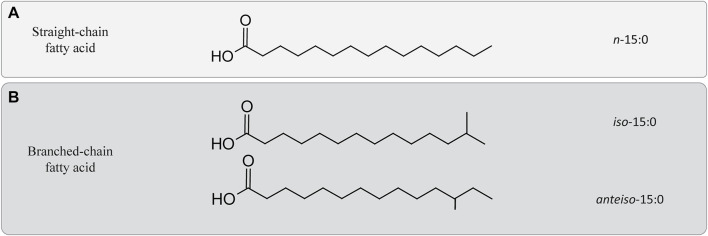
Differences between straight and branched-chain fatty acids. **(A)** Example of a straight-chain fatty acid containing 15 carbons. **(B)** Two branched-chain fatty acids chain containing 15 carbons. *Iso*-15:0 refers to configuration of the acyl chain containing a methyl group attached on the penultimate, while *anteiso-*15:0 refers to a similar configuration in which the methyl group would be attached to the antepenultimate carbon of the acyl chain.

Another acyl change modification of the bacterial/eukaryotic fatty acids that has been related to the homeoviscous adaptation under HHP is related to an increase in the degree of branching of the esterified fatty acids (Mostofian et al., 2019). In piezophile and piezotolerant strains, the most commonly occurring branched-chain fatty acids are *iso* and *anteiso* fatty acids (Figure 2). *Iso* and *anteiso* fatty acids are usually found in Gram-positive and sulfate-reducing bacteria (Kaneda, 1991), but they have also been reported in the membrane of some piezophiles (Fang and Kato, 2007). For example, *Shewanella benthica* and *P. profundum* both synthesize *iso* fatty acids under HHP (Allen et al., 1999; Fang and Kato, 2007). Consequently, changes in their relative abundance have been previously interpreted as a strategy to deal with HHP (Bartlett, 1999). As an example, the gram-positive *Clostridium paradoxum* increased its relative abundance of *iso* and *anteiso* fatty acids at increasing hydrostatic pressure at a given growth temperature (Table 1; Li et al., 1993; Scoma et al., 2019). This strategy in *C. paradoxum* coincided with an increase of the proportion of shorter carbon chain fatty acids in the membrane with increasing hydrostatic pressure to increase the fluidity of the membrane (Scoma et al., 2019). Nevertheless, other piezotolerant bacteria, e.g., *Colwellia piezophila, Moritella yayanosii,* and *Psychromonas kaikoae,* do not produce branched-chain fatty acids (Table 1)*,* revealing that branched-chain fatty acids solely are not required to achieve piezophily for some microbial groups (Kaneda, 1991).

### 3.2 Polar head groups

Polar head groups attached to the glycerol backbone, both in eukaryotic/bacterial lipids, confer specific structural characteristics, such as anchorage of proteins or curvature to the membrane (Castell, 2019). Head group polarity is also a key factor to regulate membrane packing. Zwitterionic lipids, which contain an equal amount of negative and positive charges, are expected to be packed more tightly than lipids with a net positive or negative charge. Thus, polar headgroup have an important effect on the packing and in the curvature of the membrane, and can play an important role for homeoviscous adaptation.

The membrane polar head groups can be diverse, but the most common ones are serine, ethanolamine, glycerol, choline, and myo-inositol, which are found in the phospholipids in all three domains of life (Sohlenkamp and Geiger, 2015). Most of the studies that have evaluated changes in the cell membrane upon environmental stimuli, including HHP, have focused on changes in the core lipid, while little is known of how the polar head groups change in these conditions. Nonetheless, a study by Kaneko et al. (2000) reported an increase in the relative abundance of intact polar lipids (IPLs) with the phosphatidylethanolamine (PE) head group with increasing pressure in a piezotolerant strain of *Pseudomonas* sp. isolated from the deep sea and grown at elevated temperature and HHP (Canganella et al., 2000). Another study also reported an increase in the relative abundance of IPLs with phosphatidylcholine (PC) head group for the deep-sea bacterium *Photobacterium profundum* grown at HHP and low temperature (Allen et al., 1999). In contrast, a recent study reported a decrease of the relative abundance of PC IPLs at HHP in two strains of the family Marinifilaceae of the phylum Bacteroidetes (Yadav et al., 2020). Yadav et al. (2020) also observed opposing changes in the polar head group distribution upon HHP for different analyzed strains of the same genus, with PE IPLs either increasing or decreasing at HHP, concomitantly with an increase or decrease of the ornithine lipid (OL). (Yadav et al., 2020). Therefore, it is likely that polar head group modifications upon HHP are not a universal feature. In general, all the studies on the membrane adaptation upon HHP suggest that the membrane lipid response is a combination of changes on both the polar head group and the core lipids (fatty acids) as a whole, and that the nature and the direction of this change is highly dependent on the taxonomy of the strains under study.

## 4 Hopanoids and sterols

Apart from membrane lipids, other lipid-based components are part of the membrane and act as regulators modifying the permeability of the membrane by increasing its rigidity and decreasing its permeability, being these hopanoids and sterols.

Hopanoids are a diverse group of pentacyclic triterpenoids mainly produced by bacteria. Their functionalized derivatives are referred as bacteriohopanepolyols (BHPs). The structure of hopanoids resemble the one of sterols (tetracyclic triterpenoids) found mostly in eukaryotic membranes. Previous studies have been shown that hopanoids have a similar location and function than sterols (Joung et al., 2008). Both sterols and hopanoids are derived from the same precursor, squalene, which is one of the products of the isoprenoid biosynthetic pathways (Kannenberg and Poralla, 1999; Micera et al., 2020). Sterols have also been observed in a few bacterial species of aerobic methanotrophs, myxobacteria and planctomycetes and members of the Bacteroidetes. Nevertheless, these have a very low structural complexity in comparison with those of eukaryotes.

The effect of HHP on the composition and/or abundance of sterols or hopanoids in cell membranes is poorly constrained. Two piezotolerant Marinifiliceae from the Black Sea possess BHPs biosynthetic genes, however the conditions inducing the productions of those hopanoids have not yet been identified (Yadav et al., 2020). A study by Abe, (2021), evaluated the effect of HHP on the growth of the yeast *Saccharomyces cerevisiae* by using functional genomics and transcriptomics analyses, concluding that mutations causing a decrease in the abundance of the sterol ergosterol in their membrane, made the cells more sensitive to HHP and to low temperature (Abe, 2021). More studies on the changes in membrane lipids in sterol and hopanoid-producers need to be conducted to further clarify if an increase of these membrane regulators upon HHP is a universal feature or not.

## 5 Adaptation to HHP in archaeal membrane lipids

**FIGURE 3:**
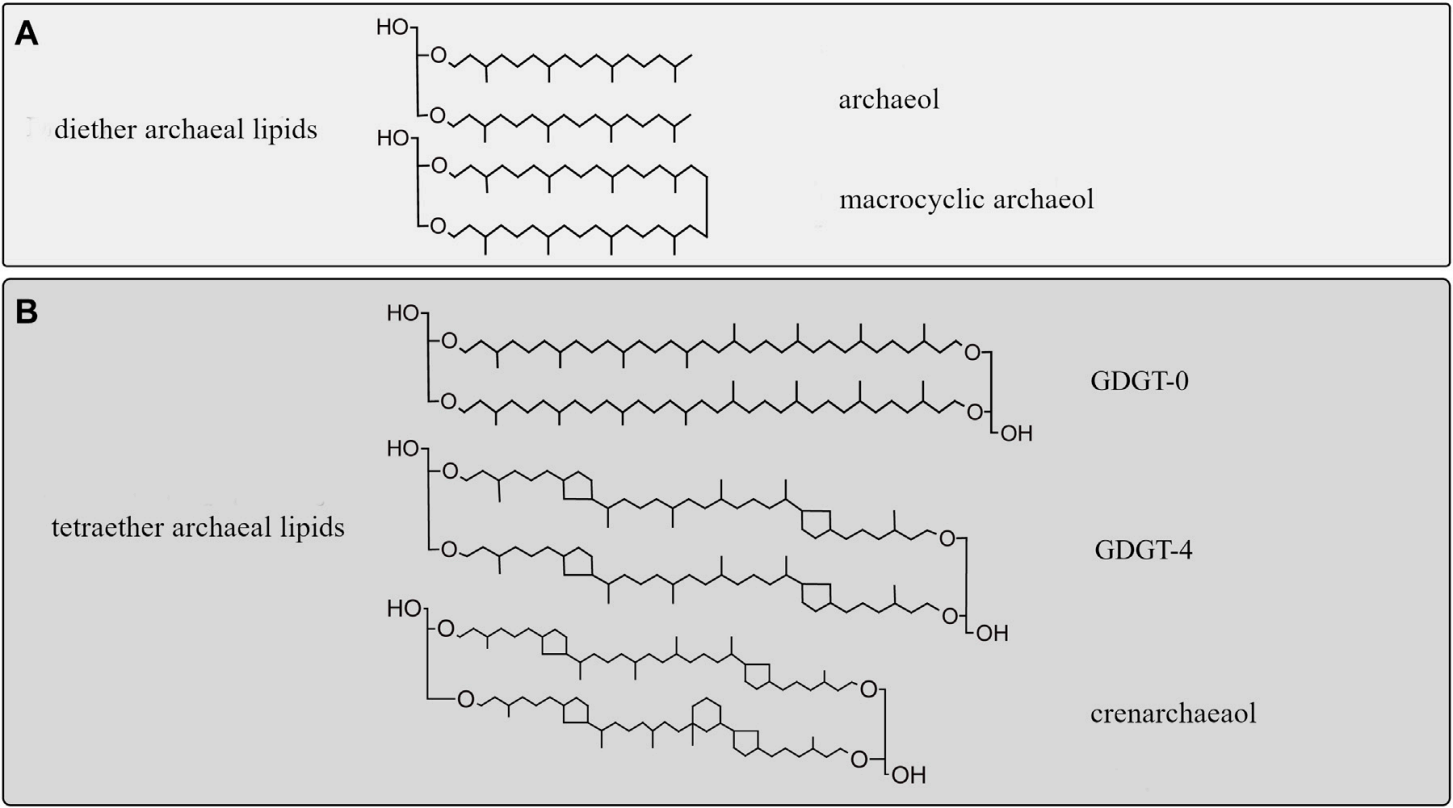
Diversity of isoprenoid chains in archaeal membrane lipids. **(A)** Archaeol (diether) membrane lipids encountered in piezophiles. **(B)** Diversity of archaeal tetraether lipids with and without moieties. Top: GDGT-0, with no cyclopentane moieties. Middle: GDGT-4 containing four cyclopentane rings, bottom: crenarchaeol, containing four cyclopentane rings and a cyclohexane ring (Damsté et al., 2002; Holzheimer et al., 2021).

Archaeal membrane lipids are based on ether-bonded isoprenoid chains with modifications, such as methylations, hydroxy groups covalent bonds, and ring moieties, the abundance of which has been related to membrane fluidity adaptation to compensate for environmental changes (Figure 3; Siliakus et al., 2017; Jebbar et al., 2015).

Membrane adaptation under HHP has only been studied in two archaeal species; in *Methanococcus jannaschii*, a thermophilic methanogen, and in *Thermococcus barophilus*, a hyperthermophile piezophile [Table 1; Kaneshiro and Clark, 1995; Cario et al., 2015)]. In both species, the ratio of diether to GDGT membrane lipids increased when they were grown at higher than optimal pressure (Cario et al., 2015). This change would theoretically result in a bulkier membrane with higher lateral mobility and lower bending rigidity (Shinoda et al., 2005). In *M. jannaschii*, this transition to diether lipid results in a strong increase of macrocyclic archaeol at the expense of GDGT-0 and archaeol (Figure 3A). The increase of macrocyclic molecules in the membrane results in a tightly packed membrane, and prevents potential leakage of solute, proton or water (Jebbar et al., 2015). Although the studies on *M. jannaschii* and *T. barophilus* point to similar adaptive strategies, the lack of additional study on piezophile or piezotolerant archaea does not allow to identify specific adaptive response to HHP in archaea.

## 6 Methodological challenges of studying the effect of HHP on the membranes of marine microbes

To gain more insight into the potential contained in piezophiles, isolation and cultivation of piezophilic microorganisms under laboratory conditions is essential. Nevertheless, the maintenance of HHP during sampling and further cultivation is rather challenging and advancements in this regard have been only possible as a result of developments in specific equipments (Bartlett, 2002; Garel et al., 2019). These constraints have severely biased the types of piezophiles available in culture from which their physiology has been investigated, with a preference for oxygen-consuming (aerobic) microbes or piezophilic (hyper)thermophiles collected from hydrothermal vents. In this regard, most of the studied piezophiles are bacteria, while little is known about piezophilic archaea due to their slower growth rate and because they either consume or generate gasses out of their metabolism.

Methods to extract and analyze microbial membranes in general, and membrane lipids in particular, have been widely optimized (Carrasco-Pancorbo et al., 2009; Aldana et al., 2020). Nevertheless, the main problem is to assure that microbial membranes do not change upon decompression of the cultures or enrichments prior to analysis. Previous studies have assessed the effect of decompression on microbial growth, cell mobility and morphology, but the effect on membrane composition has yet to be examined (Cario et al., 2022a). Similar caveats have been faced when studying changes in microbial gene expression upon HHP, and a solution to it has been to fix samples while they are under HHP (Feike et al., 2012). To the best of our knowledge, no studies have been performed with fixed samples under HHP for the purpose of lipid analysis, and there is no knowledge regarding how membranes, or membrane lipids specifically, would be affected by the use of fixatives that would arrest cell activity. Still, these kinds of studies would be essential to discard changes in the microbial membrane during decompression which would be independent on those caused by HHP.

Among future challenges are the live observation of changes in the permeability or integrity of the cell membrane upon changes in hydrostatic pressure by using live-cell imaging methods. In this regard, previous studies have adjusted microscope setups to be able to perform high-resolution quantitative imaging of live cells under HHP (Bourges et al., 2020). In addition, the development of microfluidic chip now allows to observe phenotype change of microbes under HHP in real-time, without depressurization (Cario et al., 2022b). Another way to determine potential changes in the membrane lipids upon HHP, might be to determine the microbial genomic potential to synthesize unsaturated and branched-chain fatty acids, which have been seen to increase as a response to HHP. Lipid biosynthetic pathways and their protein-coding genes are, in some cases, quite constrained and it is possible to detect, determine the diversity, and or the expression of specific genes as a proxy of the diversity of microorganisms producing a given lipid, or the up and downregulation of the gene expression leading to it (Pearson et al., 2007; Villanueva et al., 2014). This approach has proven to be very useful when applied to environmental settings to better constrain the distribution and/or abundance of producers of specific membrane lipids (Kim et al., 2016; Besseling et al., 2018).

## 7 Conclusions and future challenges

In conclusion, piezophiles and piezotolerant prokaryotes have multiple mechanisms to maintain the integrity of their cell membrane when challenged to grow under HHP, such as an increased degree of unsaturation or branching of fatty acids. However, the extent of those mechanisms is not fully constrained as most studies have focused solely on modifications in the acyl chain, and the impact of HHP on polar head groups, the presence of hopanoids and/or sterols, and membrane proteins remains largely unknown. Unveiling those aspects of membrane adaptations would allow to have a comprehensive picture of microbial adaptation, and possibly explain the substantial differences in fatty acid distribution found even between species of the same genus. Similarly, the limited research on the impact of the chemical composition of archaeal membrane on its physical properties does not allow at present to draw broad conclusion on archaeal adaptation, although the two piezophiles archaea studied seem to have similar adaptive strategies. This scarcity can be explained by the difficulty of studying piezophiles and piezotolerant microbes in the lab. Developments of accessible and low-maintenance high pressure incubators would allow to culture and identify more piezophiles and piezotolerant strains, and potentially highlight specific adaptations to HHP.

Another challenge for high pressure incubators and samplers is the decompression process: the transition to atmospheric pressure is likely to impact the membrane composition—the extent of this impact being still unknown. Alternatively, molecular omics methods present a way to circumvent the culturing difficulty and allow to predict the potential of a microbial community. Such studies require well-characterized lipid biosynthetic pathways. Future challenges would include a combined omics study, combining lipidomics, metagenomics and proteomics, to fully assess the specific membrane lipid adaptations of environmental microbial communities.

## References

[B1] Abe F. (2021). Molecular responses to high hydrostatic pressure in eukaryotes: Genetic insights from studies on saccharomyces cerevisiae. Biology 10 (12), 1305–1315. 10.3390/biology10121305 34943220 PMC8698847

[B2] Aldana J. Romero-Otero A. Cala M. P. (2020). Exploring the lipidome : Current lipid extraction techniques for mass spectrometry analysis. Metabolites 10 (6), 231–236. 10.3390/metabo10060231 32503331 PMC7345237

[B3] Allen E. E. Facciotti D. Bartlett D. H. (1999). Monounsaturated but not polyunsaturated fatty acids are required for growth of the deep-sea bacterium *Photobacterium profundum* SS9 at high pressure and low temperature. Appl. Environ. Microbiol. 65 (4), 1710–1720. 10.1128/aem.65.4.1710-1720.1999 10103272 PMC91242

[B4] Bartlett D. H. (1999). Microbial adaptations to the psychrosphere/piezosphere. J. Mol. Microbiol. Biotechnol. 1 (1), 93–100. 10941790

[B5] Bartlett D. H. (2002). Pressure effects on *in vivo* microbial processes. Biochimica Biophysica Acta - Protein Struct. Mol. Enzym. 1595 (1–2), 367–381. 10.1016/S0167-4838(01)00357-0 11983409

[B6] Bartlett D. H. Ferguson G. Valle G. (2014). Adaptations of the psychrotolerant piezophile *Photobacterium profundum* Strain SS9. High-Pressure Microbiol. 319–337. 10.1128/9781555815646.ch18

[B7] Besseling M. A. Hopmans E. C. Boschman R. C. Sinninghe Damste J. S. Villanueva L. (2018). Benthic archaea as potential sources of tetraether membrane lipids in sediments across an oxygen minimum zone. Biogeosciences 15, 4047–4064. 10.5194/bg-15-4047-2018

[B8] Bourges A. C. Lazarev A. Declerck N. Rogers K. L. Royer C. A. (2020). Quantitative high-resolution imaging of live microbial cells at high hydrostatic pressure. Biophysjcs 118 (11), 2670–2679. 10.1016/j.bpj.2020.04.017 PMC7264842 32402241

[B9] Buckles L. K. Villanueva L. Weijers J. W. H. Verschuren D. Damste J. S. S. (2013). Linking isoprenoidal GDGT membrane lipid distributions with gene abundances of ammonia-oxidizing *Thaumarchaeota* and uncultured crenarchaeotal groups in the water column of a tropical lake (Lake Challa , East Africa ). Environ. Microbiol. 15 (9), 2445–2462. 10.1111/1462-2920.12118 23560451

[B10] Canganella F. GambAcortA A. Kato C. HoriKoshi K. (2000). Effects of hydrostatic pressure and temperature on physiological traits of *Thermococcus guaymasensis* and *Thermococcus aggregans* growing on starch. Microbiol. Res. 154 (4), 297–306. 10.1016/S0944-5013(00)80003-8 10772151

[B11] Cario A. Grossi V. Schaeffer P. Oger P. M. (2015). Membrane homeoviscous adaptation in the piezo-hyperthermophilic archaeon *Thermococcus barophilus* . Front. Microbiol. 6 (OCT), 1–12. 10.3389/fmicb.2015.01152 26539180 PMC4612709

[B12] Cario A. Larzilliere M. Nguyen O. Alain K. Marre S. (2022a). High-pressure microfluidics for ultra-fast microbial phenotyping. Front. Microbiol. 13, 866681. 10.3389/fmicb.2022.866681 35677901 PMC9168469

[B13] Cario A. Oliver G. H. Rogers K. L. (2022b). Characterizing the piezosphere : The effects of decompression on microbial growth dynamics. Front. Microbiol. 13 (MAY), 1–15. 10.3389/fmicb.2020.01023 PMC9157427 35663870

[B14] Carrasco-Pancorbo A. Navas-Iglesias N. Cuadros-Rodríguez L. (2009). From lipid analysis towards lipidomics, a new challenge for the analytical chemistry of the 21^st^ century. Part I Morder lipid analysis’ TrAC Trends Anal. Chem. 28 (3), 263–278. 10.1016/j.trac.2008.12.005

[B15] Castell M. S. (2019). Apolar lipids, the membrane adaptation toolbox of extremophiles. Lyon, France: Institut des Sciences Appliquées de Lyon. [PhD thesis] [Lyon (FR)].

[B16] Chwastek G. Surma M. A. Rizk S. Grosser D. Lavrynenko O. Rucinska M. (2020). Principles of membrane adaptation revealed through environmentally induced bacterial lipidome remodeling. Cell Rep. 32, 108165. 10.1016/j.celrep.2020.108165 32966790

[B17] Damsté J. S. S. Schouten S. Hopmans E. C. van Duin A. C. T. Geenevasen J. A. J. (2002). Crenarchaeol : The characteristic core glycerol dibiphytanyl glycerol tetraether membrane lipid of cosmopolitan pelagic crenarchaeota. J. Lipid Res. 43, 1641–1651. 10.1194/jlr.M200148-JLR200 12364548

[B18] DeLong E. F. Yayanos A. A. (1986). Biochemical function and ecological significance of novel bacterial lipids in deep-sea procaryotes. Appl. Environ. Microbiol. 51 (4), 730–737. 10.1128/aem.51.4.730-737.1986 16347037 PMC238956

[B19] Ernst R. Ejsing C. S. Antonny B. (2016). Homeoviscous adaptation and the regulation of membrane lipids. J. Mol. Biol. 428 (24), 4776–4791. 10.1016/j.jmb.2016.08.013 27534816

[B20] Fang J. Bazylinski D. A. (2008). Deep-sea piezosphere and piezophiles: Geomicrobiology and biogeochemistry. Trends Microbiol. 18 (9), 413–422. 10.1016/j.tim.2010.06.006 20663673

[B21] Fang J. Kato C. (2007). FAS or PKS, lipid biosynthesis and stable carbon isotope fractionation in deep-sea piezophilic bacteria. Badajoz: Science and Technology.

[B22] Feike J. Jurgens K. Hollibaugh J. T. Kruger S. Jost G. Labrenz M. (2012). Measuring unbiased metatranscriptomics in suboxic waters of the central Baltic Sea using a new *in situ* fixation system. ISME J. 6, 461–470. 10.1038/ismej.2011.94 21776032 PMC3260512

[B23] Garel M. Bonin P. Martini S. Guasco S. Roumagnac M. Bhairy N. (2019). Pressure-retaining sampler and high-pressure systems to study deep-sea microbes under *in situ* conditions. Front. Microbiol. 10 (APR), 1–13. 10.3389/fmicb.2019.00453 31024462 PMC6465632

[B24] Holzheimer M. Sinninghe Damste J. S. Schouten S. Havenith R. W. A. Cunha A. V. Minnaard A. J. (2021). Total synthesis of the alleged structure of crenarchaeol enables structure revision. J. Ger. Chem. Soc. 60 (32), 17504–17513. 10.1002/anie.202105384 PMC8361987 34114718

[B25] Jannasch H. W. Taylor C. D. (1984). Deep-sea microbiology. Annu. Rev. Microbiol. 38, 487–514. 10.1146/annurev.mi.38.100184.002415 6437324

[B26] Jebbar M. Franzetti B. Girard E. Oger P. (2015). Microbial diversity and adaptation to high hydrostatic pressure in deep-sea hydrothermal vents prokaryotes. Extremophiles 19, 721–740. 10.1007/s00792-015-0760-3 26101015

[B27] Joung T. H. Lee J. H. Nho I. S. Lee C. M. Lee P. M. Aoki T. (2008). A study on the pressure vessel design, structural analysis and pressure test of a 6000 m depth-rated unmanned underwater vehicle. Ships Offshore Struct. 3 (3), 205–214. 10.1080/17445300802204371

[B28] Kamimura K. Fuse H. Takimura O. Yamaoka Y. (1993). Effects of growth pressure and temperature on fatty acid composition of a barotolerant deep-sea bacterium. Appl. Environ. Microbiol. 59 (3), 924–926. 10.1128/aem.59.3.924-926.1993 16348900 PMC202211

[B29] Kaneda T. (1991). Iso- and anteiso-fatty acids in bacteria: Biosynthesis, function, and taxonomic significance. Microbiol. Rev. 55 (2), 288–302. 10.1128/mr.55.2.288-302.1991 1886522 PMC372815

[B30] Kaneko H. Takami H. Inoue A. HoriKoshi K. (2000). Effects of hydrostatic pressure and temperature on growth and lipid composition of the inner membrane of barotolerant *Pseudomonas* sp. BT1 isolated from the deep-sea. Biosci. Biotechnol. Biochem. 64, 72–79. 10.1271/bbb.64.72 10705450

[B31] Kaneshiro S. M. Clark D. S. (1995). Pressure effects on the composition and thermal behavior of lipids from the deep-sea thermophile *Methanococcus jannaschii* . J. Bacteriol. 177 (13), 3668–3672. 10.1128/jb.177.13.3668-3672.1995 7601829 PMC177081

[B32] Kannenberg E. L. Poralla K. (1999). Hopanoid biosynthesis and function in bacteria. Naturwissenschaften 86 (4), 168–176. 10.1007/s001140050592

[B33] Kato C. (2011). Distribution of piezophiles, 644–655. 10.1007/978-4-431-53898-1 Extrem. Handb.

[B34] Kawamoto J. Sato T. Nakasone K. Kato C. Mihara H. Esaki N. (2011). Favourable effects of eicosapentaenoic acid on the late step of the cell division in a piezophilic bacterium, *Shewanella violacea* DSS12, at high-hydrostatic pressures. Environ. Microbiol. 13, 2293–2298. 10.1111/j.1462-2920.2011.02487.x 21518217

[B35] Kim J-H. Villanueva L. Zell C. Sinninghe Damsté J. S. (2016). Biological source and provenance of deep-water derived isoprenoid tetraether lipids along the Portuguese continental margin, 172, 177–204. 10.1016/j.gca.2015.09.010

[B36] Lacombe H. Tchernia P. Gamberoni L. (1985). Variable bottom water in the Western Mediterranean basin. Prog. Oceanogr. 14 (C), 319–338. 10.1016/0079-6611(85)90015-1

[B37] Li Y. Mandelc L. Wiegel J. (1993). Isolation and characterization of a moderately thermophilic anaerobic alkaliphile*, Clostridium paradoxum* sp. nov. Int. J. Syst. Bacteriol. 43 (3), 450–460. 10.1099/00207713-43-3-450

[B38] Micera M. Botto A. Geddo F. Antoniotti S. Bertea C. M. Levi R. (2020). Squalene: More than a step toward sterols. Antioxidants 9 (8), 1–14. 10.3390/antiox9080688 PMC7464659 32748847

[B39] Moi I. M. Leow A. T. C. Ali M. S. M. Rahman R. N. Z. R. A. Salleh A. B. Sabri S. (2018). Polyunsaturated fatty acids in marine bacteria and strategies to enhance their production. Appl. Microbiol. Biotechnol. 102 (14), 5811–5826. 10.1007/s00253-018-9063-9 29749565

[B40] Mostofian B. Zhuang T. Cheng X. Nickels J. D. (2019). Branched-chain fatty acid content modulates structure, fluidity, and phase in model microbial cell membranes. J. Phys. Chem. 123 (27), 5814–5821. 10.1021/acs.jpcb.9b04326 31251616

[B41] Nogi Y. (2008). Bacteria in the deep sea: Psychropiezophiles. Psychrophiles Biodivers. Biotechnol. 73, 73–82. 10.1007/978-3-540-74335-4_5

[B42] Nogi Y. Hosoya S. Kato C. Horikoshi K. (2004). *Colwellia piezophila* sp. nov., a novel piezophilic species from deep-sea sediments of the Japan Trench. Int. J. Syst. Evol. Microbiol. 54 (5), 1627–1631. 10.1099/ijs.0.03049-0 15388720

[B43] Nogi Y. Hosoya S. Kato C. Horikoshi K. (2007). *Psychromonas hadalis* sp. nov., a novel piezophilic bacterium isolated from the bottom of the Japan Trench. Int. J. Syst. Evol. Microbiol. 57 (6), 1360–1364. 10.1099/ijs.0.64933-0 17551059

[B44] Nogi Y. Kato C. Horikoshi K. (1998a). *Moritella japonica* sp. nov., a novel barophilic bacterium isolated from a Japan Trench sediment. J. General Appl. Microbiol. 44, 289–295. 10.2323/jgam.44.289 12501424

[B45] Nogi Y. Kato C. Horikoshi K. (1998b). Taxonomic studies of deep-sea barophilic *Shewanella* strains and description of *Shewanella violacea* sp. nov. Archives Microbiol. 170 (5), 331–338. 10.1007/s002030050650 9818352

[B46] Nogi Y. Kato C. (1999). Taxonomic studies of extremely barophilic bacteria isolated from the Mariana Trench and description of *Moritella yayanosii* sp. nov., a new barophilic bacterial isolate. Extremophiles 3 (1), 71–77. 10.1007/s007920050101 10086847

[B47] Oger P. Cario A. (2014). The high pressure life of piezophiles. Biol. Aujourd’hui 208 (3), 193–206. 10.1051/jbio/2014023 25474000

[B48] Oger P. Jebbar M. (2011). The many ways of coping with pressure. Res. Microbiol. 161 (10), 799–809. 10.1016/j.resmic.2010.09.017 21035541

[B49] Pearson A. Flood Page S. R. Jorgensen T. L. Fischer W. W. Higgins M. B. (2007). Novel hopanoid cyclases from the environment. Environ. Microbiol. 9, 2175–2188. 10.1111/j.1462-2920.2007.01331.x 17686016

[B50] Schouten S. Hopmans E. C. Damsté J. S. S. (2013). The organic geochemistry of glycerol dialkyl glycerol tetraether lipids: A review. Org. Geochem. 54, 19–61. 10.1016/j.orggeochem.2012.09.006

[B51] Scoma A. Garrido-Amador P. Nielsen S. D. Roy H. Kjeldsen K. U. (2019). The polyextremophilic bacterium *Clostridium paradoxum* attains piezophilic traits by modulating its energy metabolism and cell membrane composition. Appl. Environ. Microbiol. 85 (15), e00802-e00819. 10.1128/AEM.00802-19 31126939 PMC6643245

[B52] Shinoda W. Shinoda K. Baba T. Mikami M. (2005). Molecular dynamics study of bipolar tetraether lipid membranes. Biophysical J. 89 (5), 3195–3202. 10.1529/biophysj.105.060962 PMC1366815 16100279

[B53] Siliakus M. F. van der Oost J. Kengen S. W. M. (2017). Adaptations of archaeal and bacterial membranes to variations in temperature, pH and pressure. Extremophiles 21 (4), 651–670. 10.1007/s00792-017-0939-x 28508135 PMC5487899

[B54] Simonato F. Campanaro S. Lauro F. M. Vezzi A. D'Angelo M. Vitulo N. (2006). Piezophilic adaptation: A genomic point of view. J. Biotechnol. 126 (1), 11–25. 10.1016/j.jbiotec.2006.03.038 16780980

[B55] Small D. M. (1984). Lateral chain packing in lipids and membranes. J. Lipid Res. 25 (13), 1490–1500. 10.1016/s0022-2275(20)34422-9 6530598

[B56] Sohlenkamp C. Geiger O. (2015). Bacterial membrane lipids: Diversity in structures and pathways. FEMS Microbiol. Rev. 40 (1), 133–159. 10.1093/femsre/fuv008 25862689

[B57] Tamegai H. Ota Y. Haga M. Fujimori H. Kato C. Nogi Y. (2011). Piezotolerance of the respiratory terminal oxidase activity of the piezophilic *Shewanella violacea* DSS12 as compared with non-piezophilic *Shewanella* species. Biosci. Biotechnol. Biochem. 75 (5), 919–924. 10.1271/bbb.100882 21597190

[B58] Usui K. Hiraki T. Kawamoto J. Kurihara T. Nogi Y. Kato C. (2012). Eicosapentaenoic acid plays a role in stabilizing dynamic membrane structure in the deep-sea piezophile *Shewanella violacea*: A study employing high-pressure time-resolved fluorescence anisotropy measurement. Biochimica Biophysica Acta - Biomembr. 1818 (3), 574–583. 10.1016/j.bbamem.2011.10.010 22037146

[B59] Vargas-Yáñez M. Garcia-Martinez M. Moya F. Balbin R. Lopez-Jurado J. Serra M. (2017). Updating temperature and salinity mean values and trends in the Western Mediterranean: The RADMED project. Prog. Oceanogr. 157 (AUG), 27–46. 10.1016/j.pocean.2017.09.004

[B60] Villanueva L. del Campo J. Geyer R. (2010). Intact phospholipid and quinone biomarkers to assess microbial diversity and redox state in microbial mats. Microb. Ecol. 60 (1), 226–238. 10.1007/s00248-010-9645-2 20237775

[B61] Villanueva L. Rijpstra W. I. C. Schouten S. Damste J. S. S. (2014). Genetic biomarkers of the sterol-biosynthetic pathway in microalgae. Environ. Microbiol. Rep. 6 (1), 35–44. 10.1111/1758-2229.12106 24596261

[B62] Wang F. Xiao X. Ou H. Y. Gai Y. (2009). Role and regulation of fatty acid biosynthesis in the response of *Shewanella piezotolerans* WP3 to different temperatures and pressures. J. Bacteriol. 191 (8), 2574–2584. 10.1128/JB.00498-08 19201790 PMC2668426

[B63] Wang J. Li J. Dasgupta S. Zhang L. Golovko M. Y. Golovko S. A. (2014). Alterations in membrane phospholipid fatty acids of gram-positive piezotolerant bacterium *Sporosarcina* sp. DSK25 in response to growth pressure. Lipids 49 (4), 347–356. 10.1007/s11745-014-3878-7 24595512

[B64] Xiao X. Wang P. Zeng X. Bartlett D. H. Wang F. (2007). *Shewanella psychrophila* sp. nov. and *Shewanella piezotolerans* sp. nov., isolated from west Pacific deep-sea sediment. Int. J. Syst. Evol. Microbiol. 57 (1), 60–65. 10.1099/ijs.0.64500-0 17220442

[B65] Yadav S. Villanueva L. Bale N. Koenen M. Hopmans E. C. Damste J. S. S. (2020). Physiological, chemotaxonomic and genomic characterization of two novel piezotolerant bacteria of the family *Marinifilaceae* isolated from sulfidic waters of the Black Sea. Syst. Appl. Microbiol. 43 (5), 1–10. 10.1016/j.syapm.2020.126122 32847788

[B66] Yano Y. NakAyAmA A. Ishihara K. Saito H. (1998). Adaptive changes in membrane lipids of barophilic bacteria in response to changes in growth pressure. Appl. Environ. Microbiol. 64 (2), 479–485. 10.1128/aem.64.2.479-485.1998 16349499 PMC106069

